# Supplementary material for: Angiopoietin1 Deficiency in Hepatocytes Affects the Growth of Colorectal Cancer Liver Metastases (CRCLM)
Source: Cancers (Basel). 2019 Dec 20;12(1):35. doi: 10.3390/cancers12010035 (PMC7016878; doi:10.3390/cancers12010035)
Supplement: Supplementary file 1 [file cancers-12-00035-s001.pdf]

Supplementary material

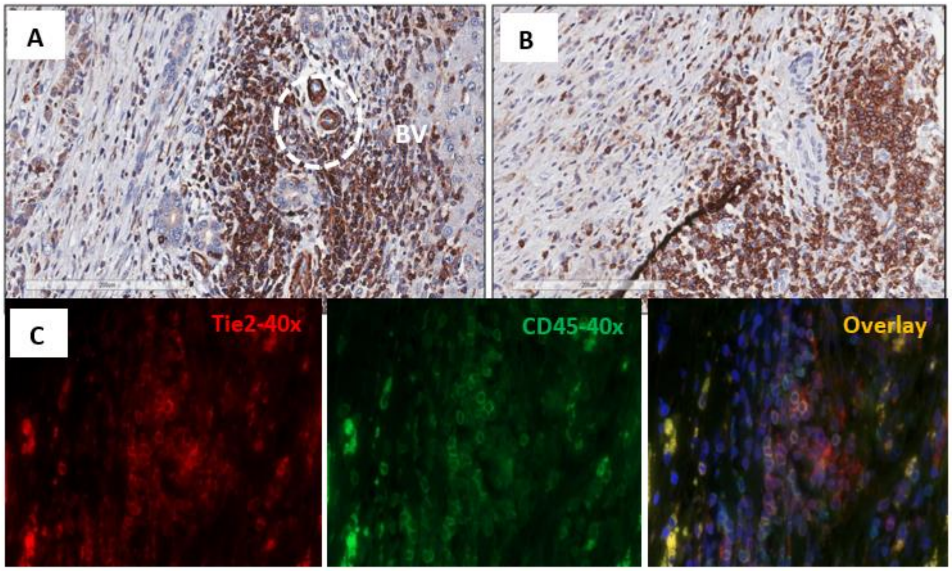

Figure S1. IHC staining and Immunofluorescent co-staining of Chemonaïve DHGP lesions. IHC staining for (**A**) Tie2 and (**B**) CD45 in serial sections of human chemonaïve DHGP lesions. (**C**) Immunofluorescent co-staining of Tie2 (red) and CD45 (green). BV-blood vessel.

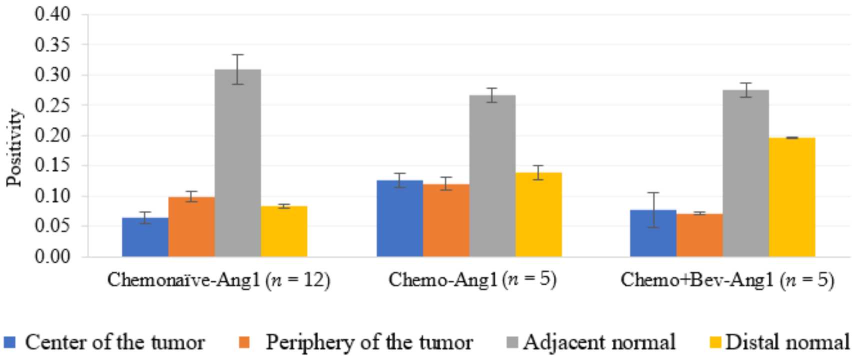

Figure S2. Expression and quantification of Ang1 expression in human chemonaïve, chemo and chemo plus Bev RHGP lesions. (**A**) the expression of Ang1 in distal normal of chemo and chemo plus Bev treated RHGP lesions. (**B**) Aperio software that quantified the positivity of the Ang1 staining of chemonaïve in comparison with chemo and chemo plus Bev of RHGP lesions. DN- distal normal, data are represented as the mean +/− SEM.

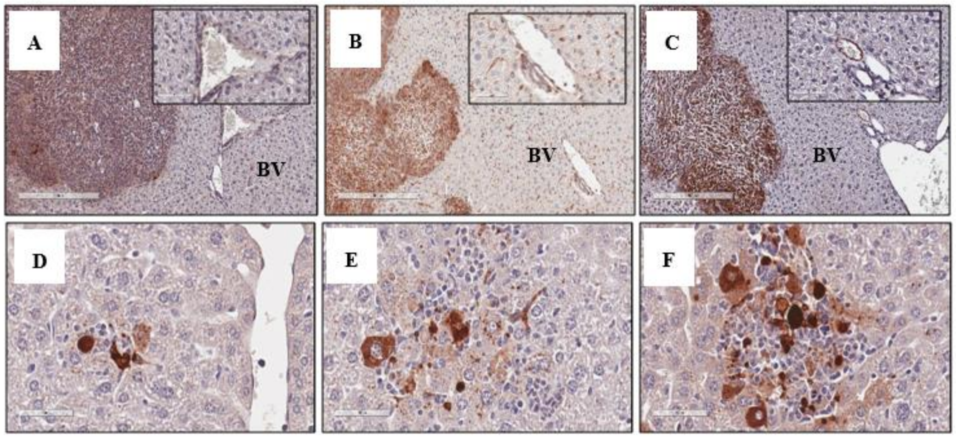

Figure S3. IHC staining of (**A**) Ang1, (**B**) Ang2 and (**C**) Tie2 expressions in RHGP mouse tumor developed from MC-38 cells metastasized into control mouse liver. (**D–F**) Ang1 IHC staining in hepatocytes surrounding tumor cells of control mouse. BV-Blood vessel.

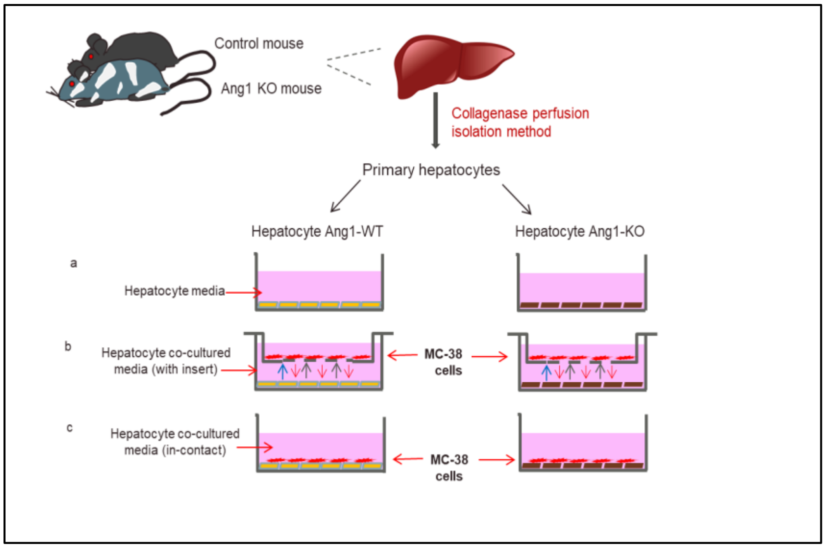

Figure S4. Schematics of different culture methods of isolated primary hepatocytes in serum free media. (**a**) Hepatocytes cultured in serum free media, (**b**) Hepatocytes co-cultured with MC-38 cells using insert, (**c**) Hepatocytes co-cultured in close contact with MC-38 cells.

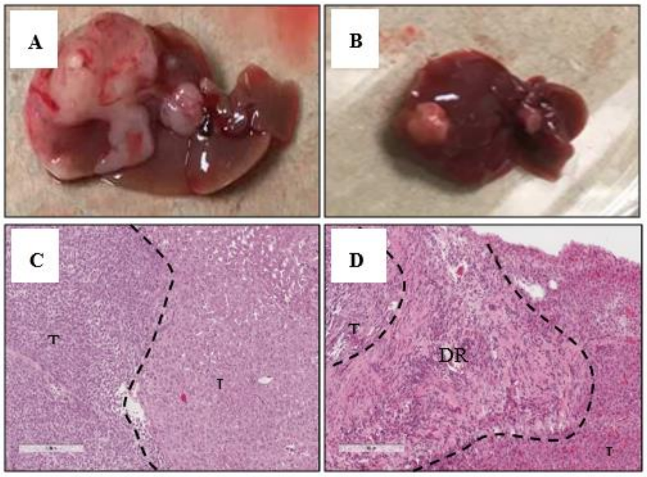

Figure S5. Liver lesions and H&E staining of transplanted RHGP tumors. Transplanted RHGP tumor grew in (**A**) control mouse and (**B**) Ang1 KO mouse. H&E staining for (**C**) RHGP tumor developed in control mouse and (**D**) DHGP tumor developed in Ang1 KO mouse. T-Tumor, L-Liver, DR-DHGP ring, dashed-lines presented the DR or the border between tumor region and livers.

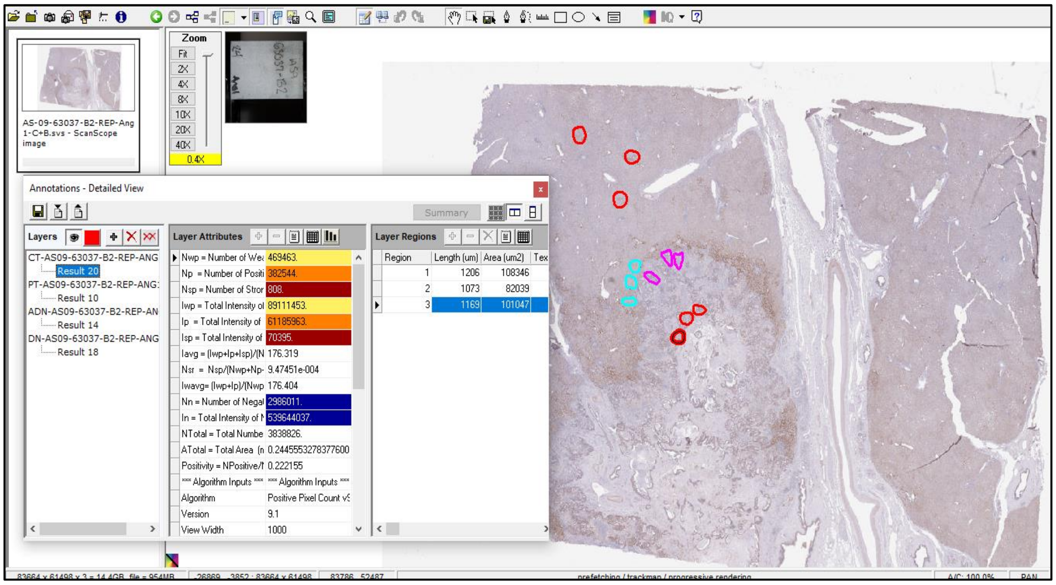

Figure S6. Aperio Image Scope software showed the stained slide scan with the annotation and analysis box displaying the results of the selected areas.

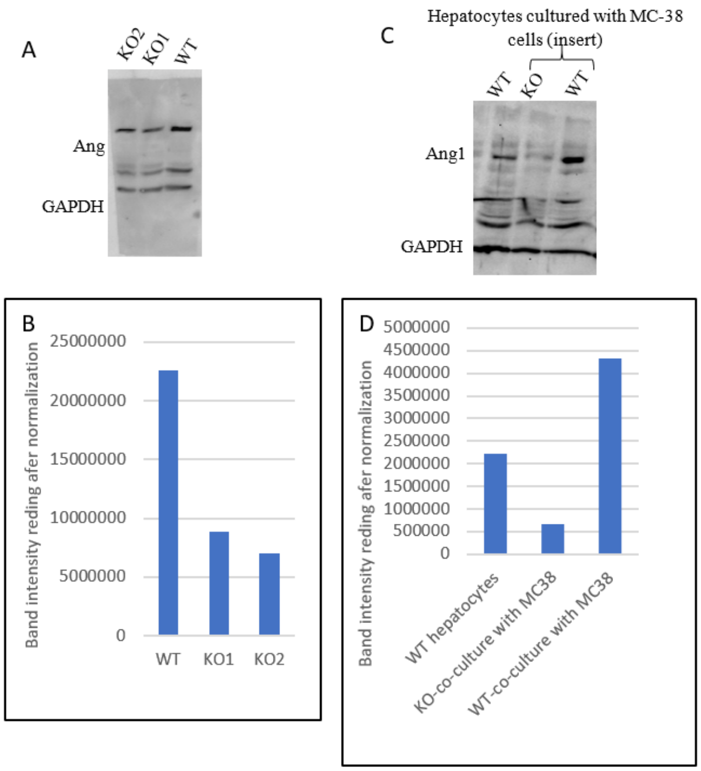

Figure S7. Western blot quantitation: Images **A** and **B** represent the quantitation of the Western blot of Figure 6B and Images **C** and **D** represent the quantitation of the Western blot of Figure 6C. We used ImageLab (Biorad) software to measure the band intensity. Both blots were normalized to their respective GAPDH signals.
